# Supplementary material for: Stable yet shifting: Early toxin dynamics in typical and atypical clownfish–anemone symbioses
Source: Toxicon X. 2026 Jul 3;31:100260. doi: 10.1016/j.toxcx.2026.100260 (PMC13356755; doi:10.1016/j.toxcx.2026.100260)
Supplement: Multimedia component 2 [file mmc2.docx]

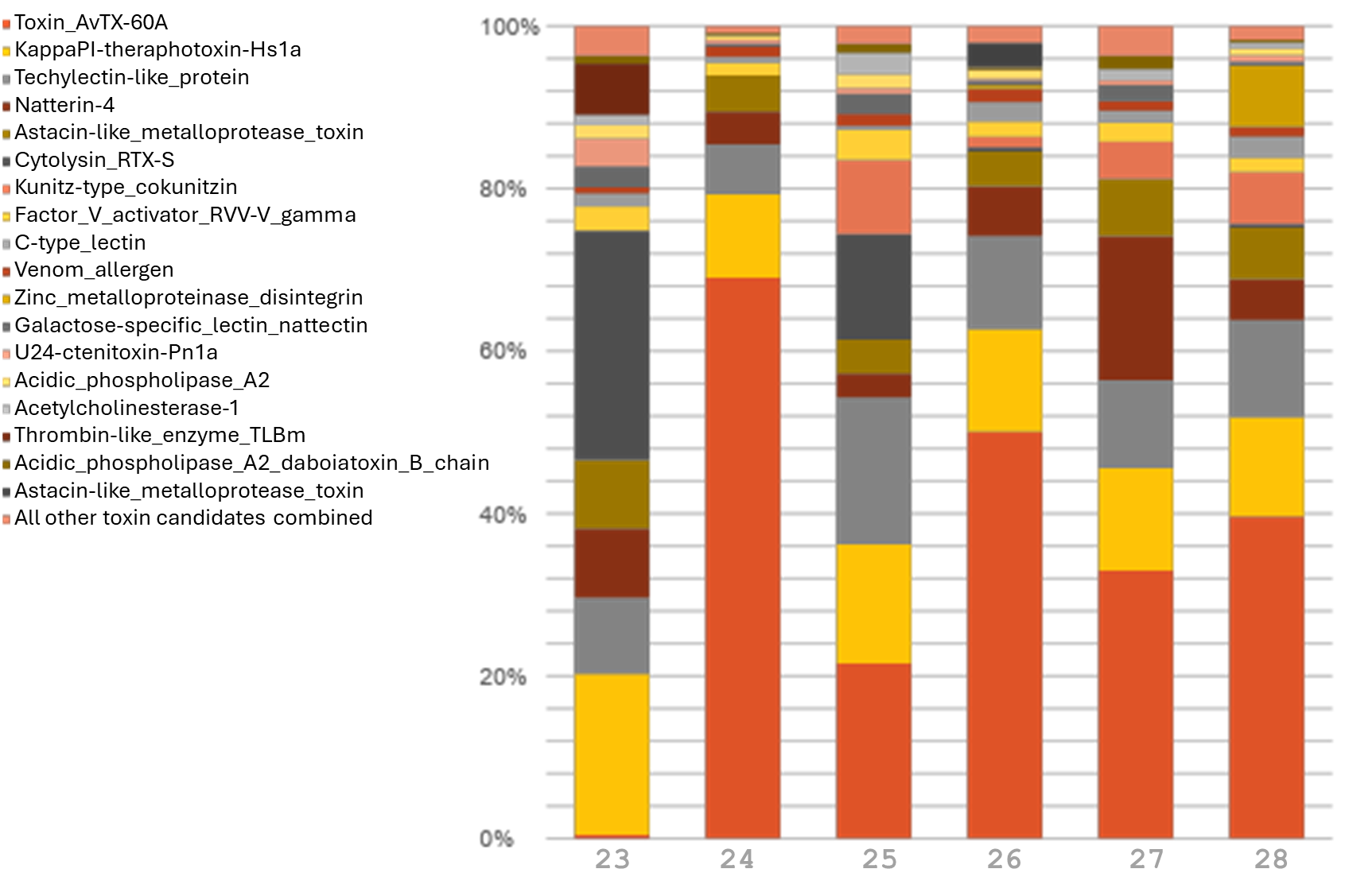


**Supplemental Figure 1.** Cumulative expression (TPM) among toxin candidates among six individual *E. quadricolor* from previously published datasets (BioProject PRJEB21970). X-axis labels correspond to the last two digits of the run ID (ERX2104223 - ERX2104228). Toxins are sorted in the figure legend, with most abundant toxins at the top of the legend, moving down as abundances decrease.

**
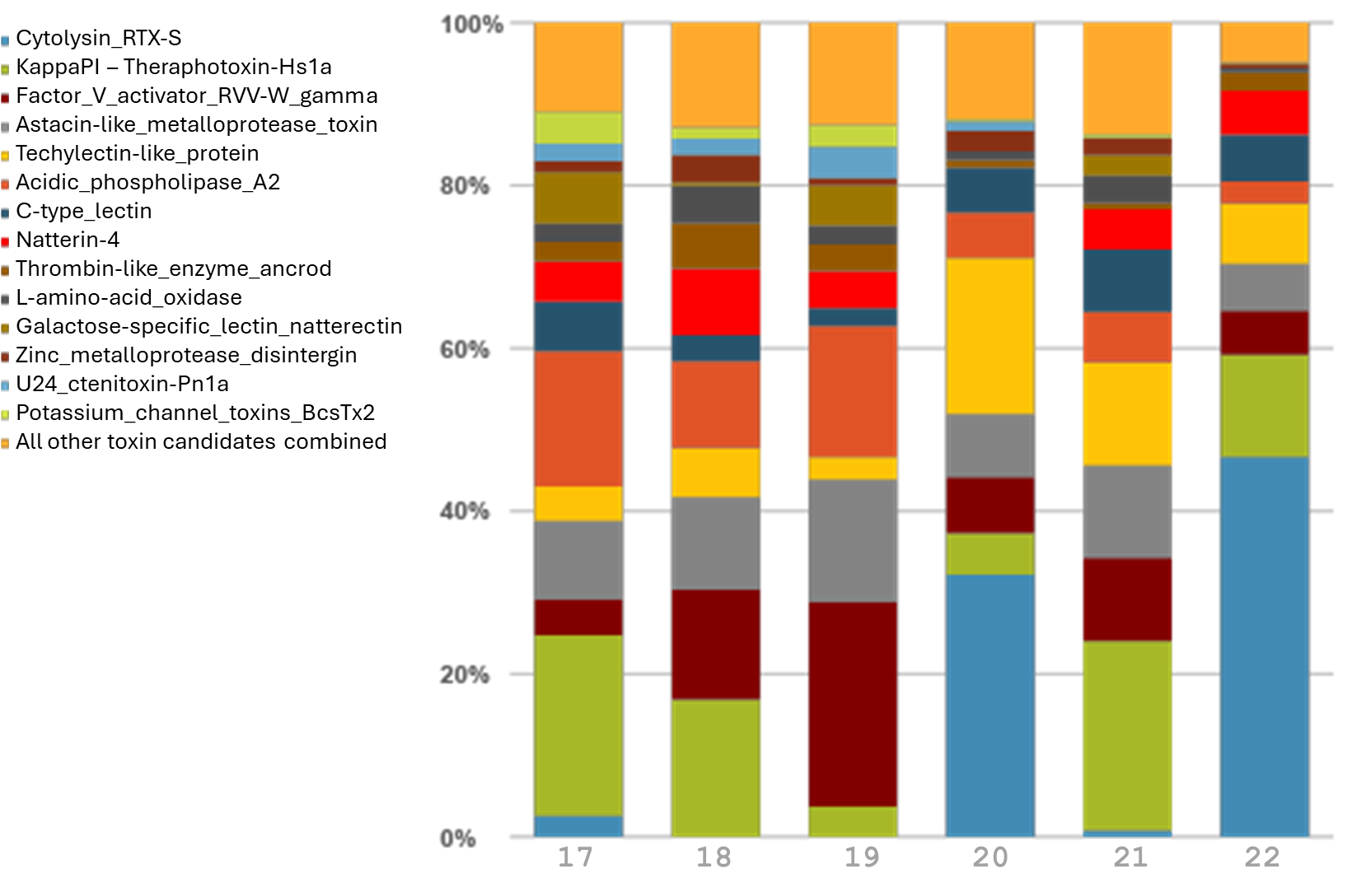
**

**Supplemental Figure 2.** Cumulative expression (TPM) among toxin candidates among six individual *C. gigantea* sea anemones from previously published datasets (BioProject PRJEB21970). X-axis labels correspond to the last two digits of the run ID (ERX2104217 - ERX2104222). Toxins are sorted in the figure legend, with most abundant toxins at the top of the legend, moving down as abundances decrease.


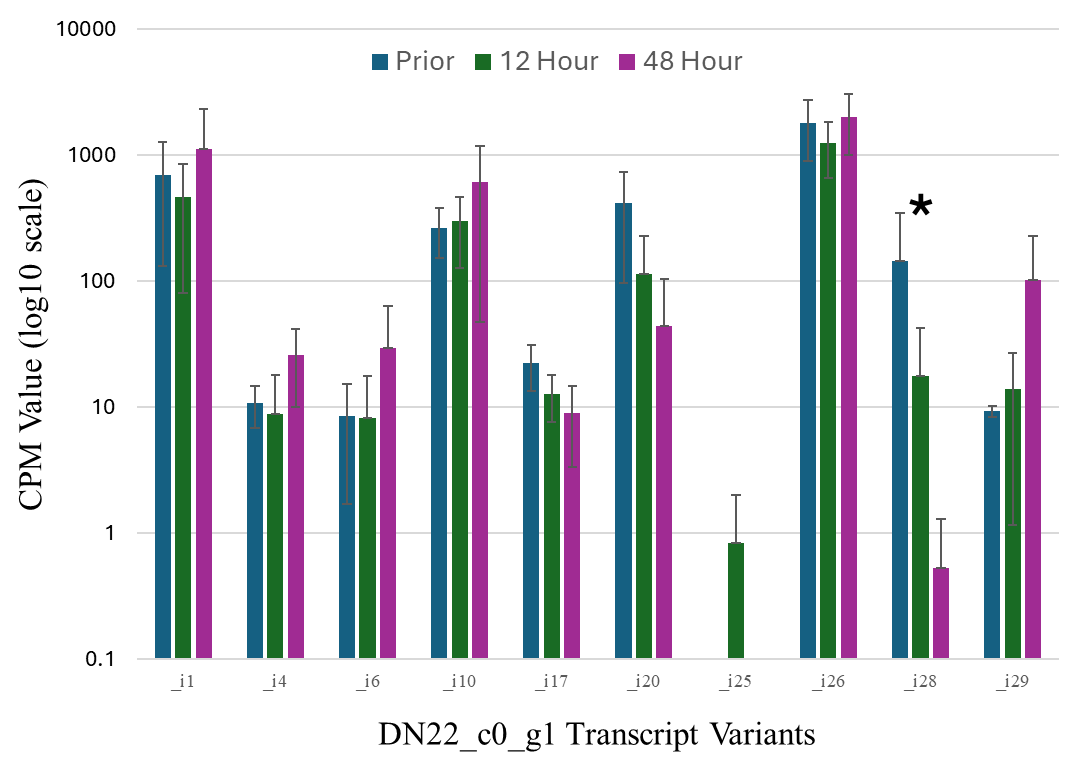


**Supplemental Figure 3.** Actinopirin expression (CPM values) across clownfish associations for various putative actinoporin transcript variants. The asterisk (*) highlights a notable fold change corresponding with strong upregulation among individual 9.

Transcript ID

1 10 20 30 40 50 60

DN22_c0_g1_i1: MSRLIVVFIVVSMICGDIALSSTKMADEKEEEDEKKNPKAIAGTVIAAAELTLAVLESVLSAIGSVNRKIAIGVANESGMDWQAKNAYYHSGTSDT**IL**PQFVPN

DN22_c0_g1_i28:MSRLIVVFIVVSMICGDIALSSTKMADEKEEEDEKKNPKAIAGTVIAAAELTLAVLESVLSAIGSVNRKIAIGVANESGMDWQAKNAYYHSGTSDT**IL**PPFVPH

DN22_c0_g1_i26:MSRLIVVFIVVSMICGDIALSSTKMADEKEEEDEKKNPKAIAGTVIAAAELTLAVLESVLSAIGSVNRKIAIGVANESGMDWQAKNAYYHSGTSDT**IL**PQFVPN

DN22_c0_g1_i25:MSRLIVVFIVVSMICGDIALSSTKMADEKEEEDEKKNPKAIAGTVIAAAELTLAVLESVLSAIGSVNRKIAIGVANESGMDWQAKNAYYHSGTSDT**IL**PQFVPN

DN22_c0_g1_i20:MSRLIVVFIVVSMICGDIALSSTKMADEKEEEDEKKNPKAIAGTVIAAAELTLAVLESVLSAIGSVNRKIAIGVANESGMDWQAKNAYYHSGTSDT**IL**PQFVPN

DN22_c0_g1_i6: MSRLIVVFIVVSMICGDIALSSTKMADEKEEEDEKKNPKAIAGTVIAAAELTLAVLESVLSAIGSVNRKIAIGVANESGMDWQAKNAYYHSGTSDT**IL**PQFVPN

DN22_c0_g1_i29:MSRLIVVFIVVSMICGDIALSSTKMADEKEEKDEKKKSAAVAGAVIDGISLTFDLLKTLLTAIGSVNRKIAIGVDNESGMQWTGRNVYYRSGTSDVHLPQYVPS

DN22_c0_g1_i17:MSRLIVVFIVVSMICGDIALSSTKMADEKEEKDEKKKSAAVAGAVIDGISLTFDLLKTLLTAIGSVNRKIAIGVDNESGIEWEGRNVYFRSGTSDVHLPQYVPS

DN22_c0_g1_i10:MSRLIVVFIVVSMICGDIALSSTKMADEKEEKDEKKKSAAVAGAVIDGISLTFDLLKTLLTAIGSVNRKIAIGVDNESGIEWEGRNVYFRSGTSDVHLPQYVPS

DN22_c0_g1_i4: MSRLIVVFIVVSMICGDIALSSTKMADEKEEKDEKKKSAAVAGAVIDGISLTFDLLKTLLTAIGSVNRKIAIGVDNESGMQWTGRNVYYRSGTSDVHLPQYVPS

Signal region ^^------ N-terminal region ------**B** **P** OO

70 80 90 100 110 120 130 140 150 160 170 180

_i1: DKALLYDGRKTHGPVARGAVGVVAYHMSDGTTLGILFSVPFDYN**F**YSNWWNIKIYEGNIEANEWMYEDLYYYANPF**EGD**NSWEYANLGEELKIAGFMTSSGEALLDIHVMR-

_i28: DKALLYDGRKTHGPVARGAVGVVAYHMSDGTTLGILFSVPFDYN**F**YSNWWNIKIYEGNIEANEWMYEDLYYYANPF**EGD**NSWEYANLGEELKIAGFMTSSGE----------

_i26: DKALLYDGRKTHGPVARGAVGVVAYHMSDGTTLGILFSVPFDYN**F**YSNWWNIKIYEGNIEANEWMYEDLYYYANPF**EGD**NSWEYANLGEELKIAGFMTSSGEALLDIHV---

_i25: DKALLYDGRKTHGPVARGAVGVVAYHMSDGTTLGILFSVPFDYN**F**YSNWWNIKIYEGNIEANEWMYEDLYYYANPF**EGD**NSWEYANLGEELKIAGFMTSSGEALLDIHVMR-

_i20: DKALLYDGRKTHGPVARGAVGVVAYHMSDGTTLGILFSVPFDYN**F**YSNWWNIKIYEGKIEASEWMYEDLYYYANPF**EGD**NSWEYANLGEELKIAGFMTSSGE----------

_i6: DKALLYDGRKTHGPVARGAVGVVAYHMSDGTTLGVLFSVPFDYN**F**YDNWWNIKIYEGKIEASEWMYEDLYYYANPF**EGD**NSWEYANLGEELKIAGFMTSSGE----------

_i29: GKALLYTARKSNGPVATGVVGVMAYYTSDGNTLAVLFSVPFDYNLYSNWWNVKIYQGKISASYSMYNELYYDANPFKGNDSWEYRNLAHGLKMEGYMNSPGEAYLRIKVSRV

_i17: GKALLYTARKSNGPVATGVVGVMAYYISNRNTLGVLFSVPYDYNLYSNWWNVKVYSGKTPADNWMYNDLYYDANPFKGNDSWEYRNLAHGLKMEGYMNSPGEAYLRIKVSRV

_i10: GKALLYTARKSNGPVATGVVGVMAYYISNRNTLGVLFSVPYDYNLYSNWWNVKIYQGKISASYSMYNELYYDANPFKGNDSWEYRNLAHGLKMEGYMNSPGEAYLRIKVSRV

_i4: GKALLYTARKSNGPVATGVVGVMAYYISNRNTLGVLFSVPYDYNLYSNWWNVKVYSGKTPADNWMYNDLYYDANPFKGNDSWEYRNLAHGLKMEGYMNSPGEAYLRIKVSRV

**P** **P** **P** S **P** **PP** OOO

**Supplemental Figure 4.** Sequence alignment of DN22_c0_g1 transcript variants. Unique amino acid residues not found in other sea anemone actinoporins adjacent to the oligomerization site are highlighted in red. The signal and N-termianl regions are indicated, with functional sites labeled as (B) site of bend when N-terminus comes into contact with the cell membrane, (P) residues involved with the POC binding site, (O) residues involved with oligomerization, (S) key sphingomyelin binding site.
